# Whole Genome Methylation Analysis of Nondysplastic Barrett Esophagus that Progresses to Invasive Cancer

**DOI:** 10.1097/SLA.0000000000002658

**Published:** 2018-01-30

**Authors:** Mark P. Dilworth, Tom Nieto, Jo D. Stockton, Celina M. Whalley, Louise Tee, Jonathan D. James, Fergus Noble, Tim J. Underwood, Michael T. Hallissey, Rahul Hejmadi, Nigel Trudgill, Olga Tucker, Andrew D. Beggs

**Affiliations:** ∗ Institute of Cancer and Genomic Science, University of Birmingham, UK; † Queen Elizabeth Hospital, Birmingham, UK; ‡ Sandwell and West Birmingham NHS Trust, Birmingham, UK; § Heart of England NHS Trust, Birmingham, UK; ¶ Cancer Sciences Unit, Faculty of Medicine, University of Southampton, Southampton, UK.

**Keywords:** Barrett esophagus, cancer genetics, esophageal cancer, methylation

## Abstract

**Objective:**

To investigate differences in methylation between patients with nondysplastic Barrett esophagus who progress to invasive adenocarcinoma and those who do not.

**Background:**

Identifying patients with nondysplastic Barrett esophagus who progress to invasive adenocarcinoma remains a challenge. Previous studies have demonstrated the potential utility of epigenetic markers for identifying this group.

**Methods:**

A whole genome methylation interrogation using the Illumina HumanMethylation 450 array of patients with nondysplastic Barrett esophagus who either develop adenocarcinoma or remain static, with validation of findings by bisulfite pyrosequencing.

**Results:**

In all, 12 patients with “progressive” versus 12 with “nonprogressive” nondysplastic Barrett esophagus were analyzed via methylation array. Forty-four methylation markers were identified that may be able to discriminate between nondysplastic Barrett esophagus that either progress to adenocarcinoma or remain static. Hypomethylation of the recently identified tumor suppressor *OR3A4* (probe cg09890332) validated in a separate cohort of samples (median methylation in progressors 67.8% vs 96.7% in nonprogressors; *P* = 0.0001, *z* = 3.85, Wilcoxon rank-sum test) and was associated with the progression to adenocarcinoma. There were no differences in copy number between the 2 groups, but a global trend towards hypomethylation in the progressor group was observed.

**Conclusion:**

Hypomethylation of *OR3A4* has the ability to risk stratify the patient with nondysplastic Barrett esophagus and may form the basis of a future surveillance program.

## Abstract 2

Supplemental Digital Content is available in the text

Esophageal adenocarcinoma (EADC) incidence is increasing^1^ and currently represents 5% of the digestive tract cancers in the UK.^2^ Overall disease survival is poor,^3^ but correlates with stage of cancer at presentation, demonstrating significant survival advantages with detection of early-stage disease.^4,5^

Barrett esophagus (BE), in which normal squamous mucosa is replaced with a metaplastic columnar phenotype, results from prolonged exposure to stomach acids and bile salts, which reflux into the esophagus causing chronic inflammation and tissue damage.^6^

The incidence of BE is increasing, largely thought to be a consequence of obesity-induced reflux disease.^7–10^ BE is associated with an increased risk of EADC,^11^ but for the majority of patients, BE will never progress beyond simple benign metaplasia.^12,13^ However, in a small number of patients, dysplasia will develop, with some progressing to EADC.^14^ The incidence of EADC in the BE population is up to 150 times greater than unaffected individuals.^12^

Although the pathological changes seen in Barrett adenocarcinoma are understood as part of a well-established metaplasia-dysplasia-carcinoma sequence,^15^ the molecular drivers are less clear.^16–18^ The current dilemma is that for patients with nondysplastic BE, there are no accurate methods for identifying the small number of patients at high risk of progression to cancer.

Surveillance practice of the Barrett patients varies widely, between some who endoscope patients each year in contrast to others who will never repeat the investigation.^19^ The ongoing UK Medical Research Council funded BOSS study aims to understanding the optimum surveillance strategy, randomizing between prospective monitoring BE patients with frequent endoscopic assessment or a “watch and wait” policy.^20^

Clearly, there is need for a method of risk stratification in these patients to facilitate a streamlined surveillance program by identifying high-risk nondysplastic BE patients. Attempts at biomarker development for stratification of high-risk BE have focused on mutational change, specifically around the role of *TP53* mutation in predicting “high-risk” disease,^21^ given its role as a driver in esophageal cancer. However, Ross-Innes et al^17^ have convincingly demonstrated the presence of pathogenic *TP53* mutations in apparently normal squamous esophageal mucosa, thus making its role in progression to invasive adenocarcinoma unclear. However, the role of epigenetic change in the pathogenesis of BE and esophageal cancer is less well-understood, but may well happen much earlier in the cancer development pathway, and, as a direct result, provide a more appropriate target for both predicting its development and potentially arresting tumorigenesis, should a suitable epigenetic modulator be identified.

Multiple methylation markers have been identified which can discriminate between high-risk and low-risk BE including *APC/p16*,^22^*MGMT*,^23^*PKP-1*,^24^*TIMP3/TERT,*^25^*RUNX3/HPP1*,^26,27^ and *AKAP12*.^28^ Agarwal et al^29^ performed a MeCIP array-based approach to compare the methylomes of progressor (n = 5) versus nonprogressing patients (n = 4). In patients who progressed to invasive adenocarcinoma, their original biopsies began either with no dysplasia, indefinite dysplasia, or low-grade dysplasia, making comparison difficult. However, subsequent analysis of the top 25 differential methylation patterns found 3 gene regions with hypermethylation amongst the progression group (*Pro_MMD2*, *Pro_ZNF358*, and *Intra_F10*), with a trend towards global hypomethylation, in keeping with other epithelial premalignant conditions.^30^ Kaz et al^31^ also found significant differences in methylation in patients with BE due to factors such as obesity, smoking, and sex, which may be responsible for some of the observed risk.

While the studies reviewed do show variation in methylation between progressive versus nonprogressive BE, the methodology has been heterogeneous, and few have conducted the study with a group of the same patients tracked over time.

The study aims to determine whether there are differences in methylation in patients between high-risk nondysplastic BE, which will progress to cancer, versus low-risk BE.

## METHODS

### Patients and Samples

Two sample cohorts were identified containing patients who either progressed to EADC from nondysplastic BE or remained with nondysplastic BE (NDBE) identified from a prospectively maintained database of patients with BE at a large district general hospital. Inclusion criteria for the study were progressing patients with nondysplastic BE who, when observed over the study period, developed EADC. Samples were only included where there was a NDBE biopsy and then histology evidence that the patient developed adenocarcinoma. Nonprogressing patients were identified from a biopsy of NDBE, which, when followed over time, never progressed beyond NDBE. To be included in this group, the patient must have been in a surveillance program for a minimum of 15 years and have serial biopsies over that period. Patients within the surveillance program had endoscopy and biopsy every 2 years.^32^ Biopsies were taken at the time of the initial surveillance endoscopy and at all subsequent endoscopies including immediately before treatment as part of their staging. They were also required to have still been alive and to have had a NDBE biopsy within 2 years of this study.

Patients were excluded from the study if BE material was only available as part of tumor-associated BE or if dysplasia was identified in any BE biopsies.

All tissue used was formalin-fixed paraffin-embedded (FFPE) samples obtained from pathology libraries and prepared by the University of Birmingham Human Biomaterials Resource Centre (ethical approval 09/H1010/75). H&E stained slides were reviewed by a consultant pathologist to ensure that the samples were BE and had no dysplasia throughout their extent. Cut 5-μM paraffin sections were mounted onto frosted slides, and macrodissection for BE was carried out. DNA extraction was then performed using Qiagen DNeasy Blood and Tissue kit following the manufacturer's protocol. Each sample of extracted DNA was then quantified and qualified by Nanodrop spectrophotometry and Qubit fluorimetry. Bisulfite conversion was performed using a Zymo DNA methylation bisulfite conversion kit following the modified Illumina Infinium protocol on 500 ng of extracted DNA.

### Methylation Arrays

The Illumina HumanMethylation 450 array, in which the methylation status of more than 485,000 individual CpG sites are examined,^33^ was used to compare sample groups (progressing NDBE vs nonprogressing NDBE). Once bisulfite converted, 1 ng of DNA was quality-controlled (Illumina FFPE QC kit) with only samples with dCt <5 being taken forward to array analysis. The resulting samples underwent repair suitable for array hybridization using the Illumina FPPE restore kit,^34^ followed by hybridization to Illumina HumanMethylation450 arrays using manufacturer's protocols and scanned on an Illumina iScan. Normalized intensity files (iDAT) were exported using GenomeStudio for downstream analysis.

### Immunohistochemistry

Immunohistochemistry (IHC) was carried out on a Leica Bond RX system using a mouse polyclonal anti-OR3A4 antibody (Abcam ab67107) at a dilution of 1:100 with a primary incubation time of 15 minutes.

Immunohistochemistry was scored on epithelial and stromal components and a composite score consisting of the sum of expression within membranous, nuclear and cytoplasmic compartments on a score of 1 to 4 was made, giving a combined maximum possible score of 12 for each compartment. Scoring was carried out by 2 independent observers blinded to progressor/nonprogressor status.

### Bioinformatics Analysis of Array Data

Bioinformatics analysis of the methylation microarrays was carried using the ChAMP package^35^ via Bioconductor/R. In brief, red/green intensity values were captured from Illumina iDAT files, background-corrected and SWAN-normalized to produce M values (further details given in supplementary methods).

M values were analyzed using a logistic regression model using Empirical Bayesian shrinkage of moderated t-statistics to correct for small sample size. Small sample size was controlled for by setting stringent false discovery rate Q values of <0.05. Identification of variable methylated sites allowed the CpG site markers to be highlighted. DNA copy number analysis was carried out using the *DNAcopy* module of ChAMP.

### Pyrosequencing Validation of Hits

Methylation-insensitive primers were designed and sourced, using Qiagen PyroMark Primer Design software v2.0. Primers were designed to flank CpG sites of interest. Illumina CG methylation probe locations were retrieved from the UCSC genome browser,^36^ and Fast-All sequence retrieved for −200 bp to +200 bp of the target CG dinucleotide. Primer design settings were optimized to design amplicons suitable for FFPE pyrosequencing, with the optimum amplicon size set to between 80 and 150 bp. All other settings were as per the standard Qiagen design parameters. Primers were ordered from Sigma-Aldrich, with the biotinylated pyrosequencing primer being purified by high-performance liquid chromatography and the remainder by desalting. Pyrosequencing PCR was performed using Qiagen PyroMark PCR Gold kit, consisting of 2 μL of bisulfite-converted DNA, 25 μL of PCR master mix, 5 μL of CoralLoad dye, 3 μL MgSO_4_, 10 μL of Q reagent, and 2.5 μL each of forward (20 mM) and reverse (20 mM) primer. Reaction conditions were determined experimentally by use of a gradient PCR for each primer pair. A typical reaction consisted of activation at 95C for 15 minutes, followed by 45 cycles of denaturation at 94°C for 30 seconds, annealing at 56°C for 30 seconds, and extension at 72°C for 30 seconds, followed by a final extension step at 72°C for 10 minutes. In addition to experimental DNA, each PCR was performed with 100% methylated DNA, 100% unmethylated DNA, and ddH_2_O as controls. Methylated and unmethylated DNA was generated in house by means of M.SSl conversion (methylated DNA) and whole genome amplification using the Qiagen Repli-G kit (unmethylated DNA). Primer sequences for validation pyrosequencing were as follows: for FGFR2 cg17337672 these were forward = AGGGGAAGGGAATTTAGGTT, reverse = [Btn]TCAATCTTCCCCCAAACAACCACT, and sequencing = GTTTAGAAGTTTTTTTTGGATTAGT; for ORA3A4 cg07863524 these were forward = GTGGTAGAAGTAGGATGAGGTGTTGATAAT, reverse = [Btn]CTTCAACTTCCTTCCCCTTACATTT, and sequencing = GGGTAGGGATGGAAGA; for OR3A4 cg09890332 these were forward = TTAAAGTGTTAGGATTATAGGTGTGAGTTA, reverse = [Btn]TTTCCCAACCCTAATCACTACTAATAAAAT, and sequencing = GGATTATAGGTGTGAGTTAT.

## RESULTS

### Patient Selection

In all, 67 patients were recruited—37 from Sandwell and West Birmingham NHS Trust (SWBNT) and 30 from University Hospital Birmingham NHS Trust (UHBFT). Of these, 20/67 progressed from nondysplastic BE and 47 did not. The age range was between 42 and 60 years, with a median age of 56 years, of which 60/67 (89.6%) were male. The median time to diagnosis of EADC in “progressor” patients was 114 months, with a range of 14–162 months. Of the patients recruited, in the SWBNT group, 6 progressors and 6 nonprogressors, and in the UHBFT, 6 progressors and 6 nonprogressors were taken forward to methylation array analysis, giving a total of 12 progressors and 12 nonprogressors. This samples size was chosen because of our previous experience with biomarker discovery in methylation arrays as a suitable size for biomarker discovery. A validation cohort of 32 patients (progressors 18, nonprogressors 14) were obtained from University Hospital Southampton. All patients included in the study had symptoms of reflux disease as a presenting symptom. For all patients in the progressor cohort (n = 30), the observed pathological disease stages at the time of resection were high-grade dysplasia (4/30, 13%), T1N0 (10/30, 33%), T1N1 (1/30, 3%), T2N0 (2/30, 7%), T2N1 (3/30,10%), T3N0 (6/30, 20%), and T3N1 (4/30, 13%). All patients with high-grade dysplasia underwent endoscopic mucosal resection and the remainder underwent esophagectomy. All patients recruited had validation pyrosequencing performed.

### Methylation Microarray Analysis

**FIGURE 1:**
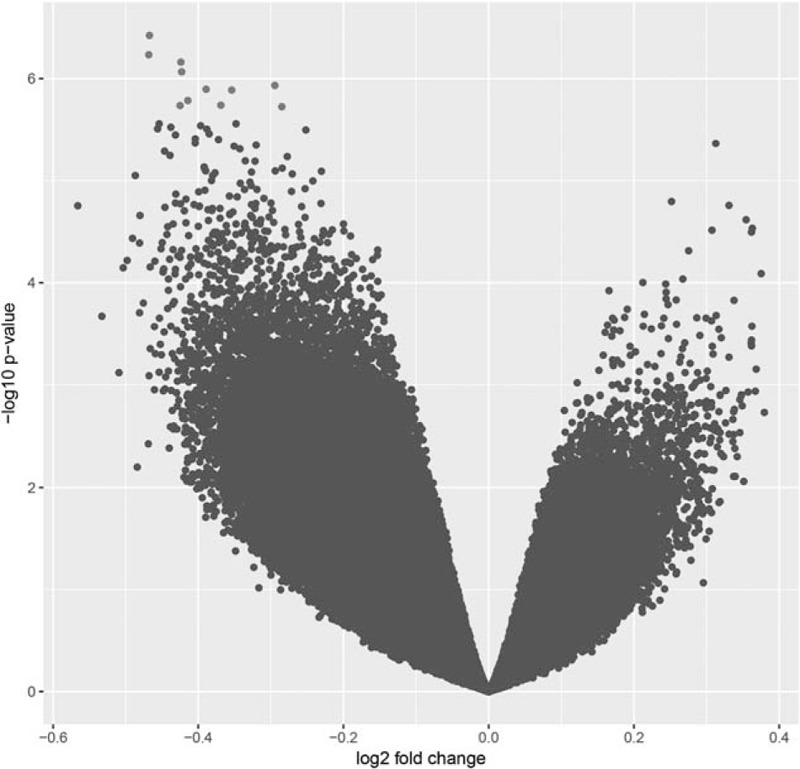
Volcano plot of probe-level methylation in progressors versus nonprogressors. Blue points = Bayes factor <5; red points = Bayes factor >5. The plot shows a leftward shift of probes towards the left, suggesting global hypomethylation.

Twenty-four samples in total were hybridized successfully to Illumina HumanMethylation450 microarrays. All arrays passed manufacturers QC as specified by metrics in Illumina GenomeStudio. Differential methylation analysis at the probe level (Table 1) revealed significant differences in methylation between progressor and nonprogressors in nondysplastic BE (Fig. 1). In all, 44 significantly [defined as Bayes factor (BF) >5, chosen as it is equivalent to a genome, wide *P* value significance of 1 × 10^−6^] differentially methylated targets were identified, the bulk being hypomethylated, with a trend towards global hypomethylation in progressor samples as demonstrated by left-shift of the Volcano plot (Fig. 1).

**TABLE 1:** Top 20 Array-identified CpG Sites With Methylation Variation Between Nondysplastic Samples Which Progress to EADC Versus Those Which Remain Static

Probe ID	*t*	*P*	Adj. *P*	Gene Name
cg09890332	−12.26	2.02E-08	0.0031	OR3A4
cg24007926	−11.05	6.84E-08	0.0032	NA
cg17337672	−10.95	7.54E-08	0.0032	FGFR2
cg02226469	−10.88	8.17E-08	0.0032	NA
cg17433294	−10.51	1.22E-07	0.0038	NMUR2
cg18479711	−10.31	1.52E-07	0.0039	HDAC4
cg09011162	−10.15	1.82E-07	0.0040	LMF1
cg19733463	−9.83	2.61E-07	0.0045	NMUR1
cg16150571	−9.64	3.26E-07	0.0045	SNORD116-22
cg24424217	−9.62	3.35E-07	0.0045	ZNF511
cg13164993	−9.57	3.52E-07	0.0045	RBP3
cg14019464	−9.53	3.70E-07	0.0045	TRIB3
cg24581378	−9.53	3.71E-07	0.0045	ZAP70
cg05230642	−9.36	4.54E-07	0.0051	SNORD115-14
cg12297814	−9.26	5.08E-07	0.0052	IGFN1
cg11231240	−9.23	5.33E-07	0.0052	NA
cg11864327	−8.96	7.40E-07	0.0063	ZFP2
cg16771467	−8.92	7.78E-07	0.0063	ATP8B1
cg17304276	−8.88	8.14E-07	0.0063	CUX2
cg11443888	−8.86	8.36E-07	0.0063	TMEM151B

Probe ID: the Illumina cg probe ID from the Illumina manifest; *T*: the *t* value (the size of the difference relative to the variation in the sample). *P* = the raw *P* value, not corrected for multiple testing; adjusted *P* = the *P* value corrected for multiple testing.

### Differential Methylation at the Probe Level

The top ranked differentially methylated probe was cg09890332 (chr17:3212495–3212495, hg19 coordinates), which tags a CpG dinucleotide −1044 bp upstream of the transcription start site of the long noncoding RNA, *OR3A4* (NRR_024128.1). The second highest ranked differentially methylated probe was cg24007926 (chr2:206842761–206842761, hg19 coordinates). This CpG dinucleotide is within a large, intragenic region, with the nearest gene being *INO80D* (INO80 complex subunit D, NM_017759.4), 15,684 bp downstream of this CpG. The third highest ranked differentially methylated probe was cg17337672 (chr10:123354172–123354172), which tags a CpG dinucleotide within intron 2 of *FGFR2* (fibroblast growth factor receptor 2, NM_000141.4).

Differentially methylated regions (DMRs) were called between progressors and nonprogressors via the *dmrLasso* function of the CHAMP software package (Table 2). Significant DMRs were found from chr2:503065–503193 (which tags an intragenic region, DMR *P* = 7.69 × 10^−4^), chr5:8217236–8217322 (which also tags an intragenic region, DMR *P* = 1.27 × 10^−3^), and chr10: 123353418–123355576, which spans a region from the 5’-UTR of *FGFR2* to the first exon within *FGFR2* (DMR *P* = 4.79 × 10^−3^).

**TABLE 2:** Table of DMRs

DMR ID	Probe ID	Probe Level Adjusted *P*	Chromosome	Gene	Start of DMR (bp)	End of DMR (bp)	Size in bp	Change in Methylation	*P* for DMR
1	cg21273584	0.014	2	NA	502999	503195	197	−34%	7.69E-04
1	cg00854591	0.045	2	NA	502999	503195	197	−16%	7.69E-04
1	cg11573608	0.014	2	NA	502999	503195	197	−27%	7.69E-04
2	cg25568703	0.047	5	NA	8216903	8217655	753	−25%	1.27E-03
2	cg25016964	0.039	5	NA	8216903	8217655	753	−22%	1.27E-03
2	cg17642708	0.021	5	NA	8216903	8217655	753	−33%	1.27E-03
3	cg10788901	0.172	10	FGFR2	123352704	123355661	2958	−27%	4.79E-03
3	cg14856220	0.044	10	FGFR2	123352704	123355661	2958	−24%	4.79E-03
3	cg17337672	0.003	10	FGFR2	123352704	123355661	2958	−47%	4.79E-03
3	cg02412684	0.031	10	FGFR2	123352704	123355661	2958	−38%	4.79E-03
3	cg06791446	0.058	10	FGFR2	123352704	123355661	2958	−13%	4.79E-03
3	cg22633036	0.581	10	FGFR2	123352704	123355661	2958	−4%	4.79E-03

We took advantage of the information provided by the 2 color Illumina Infinium chemistry to call copy number aberrations (CANs) within the regions targeted by the methylation probes using the CNA calling function of CHAMP. This did not demonstrate any recurrent copy number alterations between progressors and nonprogressors. There were no significant differences in the numbers of CNA between the 2 groups, with a median of 43 CNAs (range 24–92) in the progressors versus 44 CANs (range 30–52) in the nonprogressors (*P* = 1.0, Wilcoxon rank-sum).

Pathway methylation analysis was carried out using Database for Annotation, Visualisation and Integrated Discovery. Initially Kyoto Encylopedia of Genes & Genomes pathway analysis showed that genes associated with mitogen-activated protein kinase signaling were enriched in the dataset (*P* = 0.012). Gene ontology analysis using the UP_KEYWORDS feature showed significant enrichment for the disease mutation (*P* = 9.6 × 10^−6^), polymorphism (*P* = 1.7 × 10^−5^), glycoprotein (*P* = 3.4 × 10^−5^), and alternate splicing (*P* = 1.1 × 10^−4^) terms.

### Validation Pyrosequencing

Because of the likely biological relevance of *FGFR2*, and the data demonstrating that *OR3A4* was the top differentially methylated CpG, validation pyrosequencing was carried out on all 67 patients. Normality of distribution of methylation values was ascertained by histogram plots, in which it was found that methylation was non-normally distributed; therefore nonparametric testing was carried out.

**FIGURE 2:**
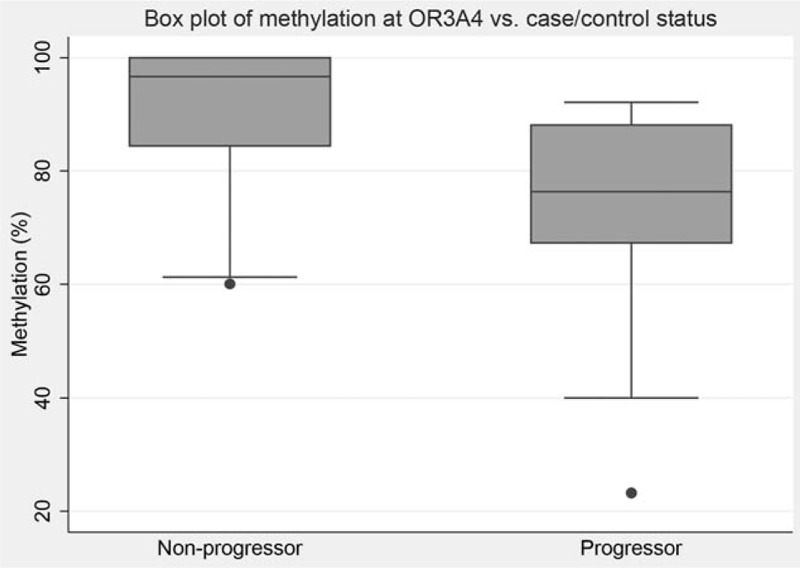
Box plot of OR3A4 methylation differentiating high-risk nondysplastic BE.

**FIGURE 3:**
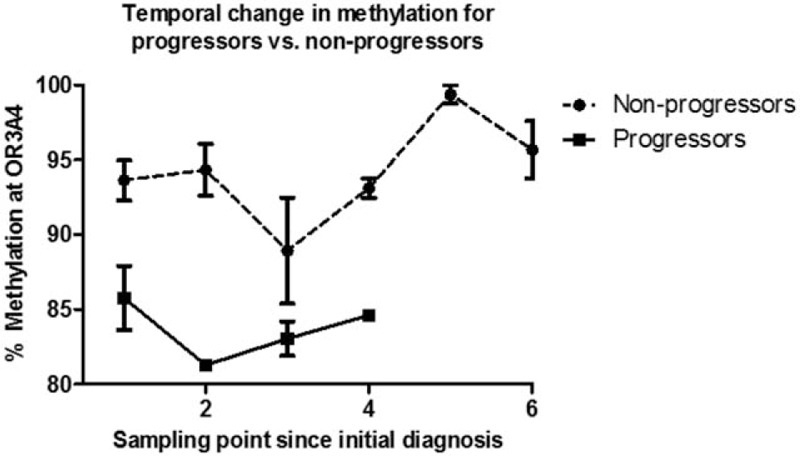
Change in methylation across temporally acquired samples for OR3A4. The y-axis shows percentage methylation at cg09890332 as observed by pyrosequencing. Time-points on the x-axis refer to the sampling points, with 1 representing the initial baseline endoscopy and subsequent visits referred by increasing numbers (surveillance intervals are variable).

For OR3A4 cg09890332, median methylation was 67.8% [interquartile range (IQR) 12.1] in progressors versus 96.7% (IQR 16.1) in nonprogressors (*P* = 0.0001, *z* = 5.158; Wilcoxon rank-sum test) (Fig. 2). The pyrosequencing assay design used covered 2 additional CpG +4 bp and +10 bp downstream of cg09890332. Median methylation in these was 66.8% and 59.7% in progressors versus 75.0% and 68.1% in nonprogressors (*P* = 0.0280 and 0.0368, *z* = 2.197 and 2.088, Wilcoxon rank-sum test). To investigate whether this phenomenon was localized to this region or was a gene-wide phenomenon, an additional pyrosequencing assay was designed based on probe ID cg07863524 (chr17:3213471–3213471), which is +976 bp downstream from cg09890332 and −68 bp from the transcription start site of OR4A4. This demonstrated that median methylation was 62.2% in progressors and 56.7% in nonprogressors (*P* = 0.600, *z* = −0.524; Wilcoxon rank-sum test). A temporal analysis of change in methylation of cg09890332 over time is shown in Fig. 3, showing that the difference between methylation levels at initial biopsy is static between progressors and nonprogressors, and that the difference is maintained over time and is detectable for an extended period of time before diagnosis of EADC.

We then validated cg17337672 within FGFR2, finding that median methylation was 83.4% in progressors versus 82.2% in nonprogressors (*P* = 0.51, *z* = 0.653; Wilcoxon rank-sum test).

### Expression of OR3A4 in Progressors Versus Nonprogressors

**FIGURE 4:**
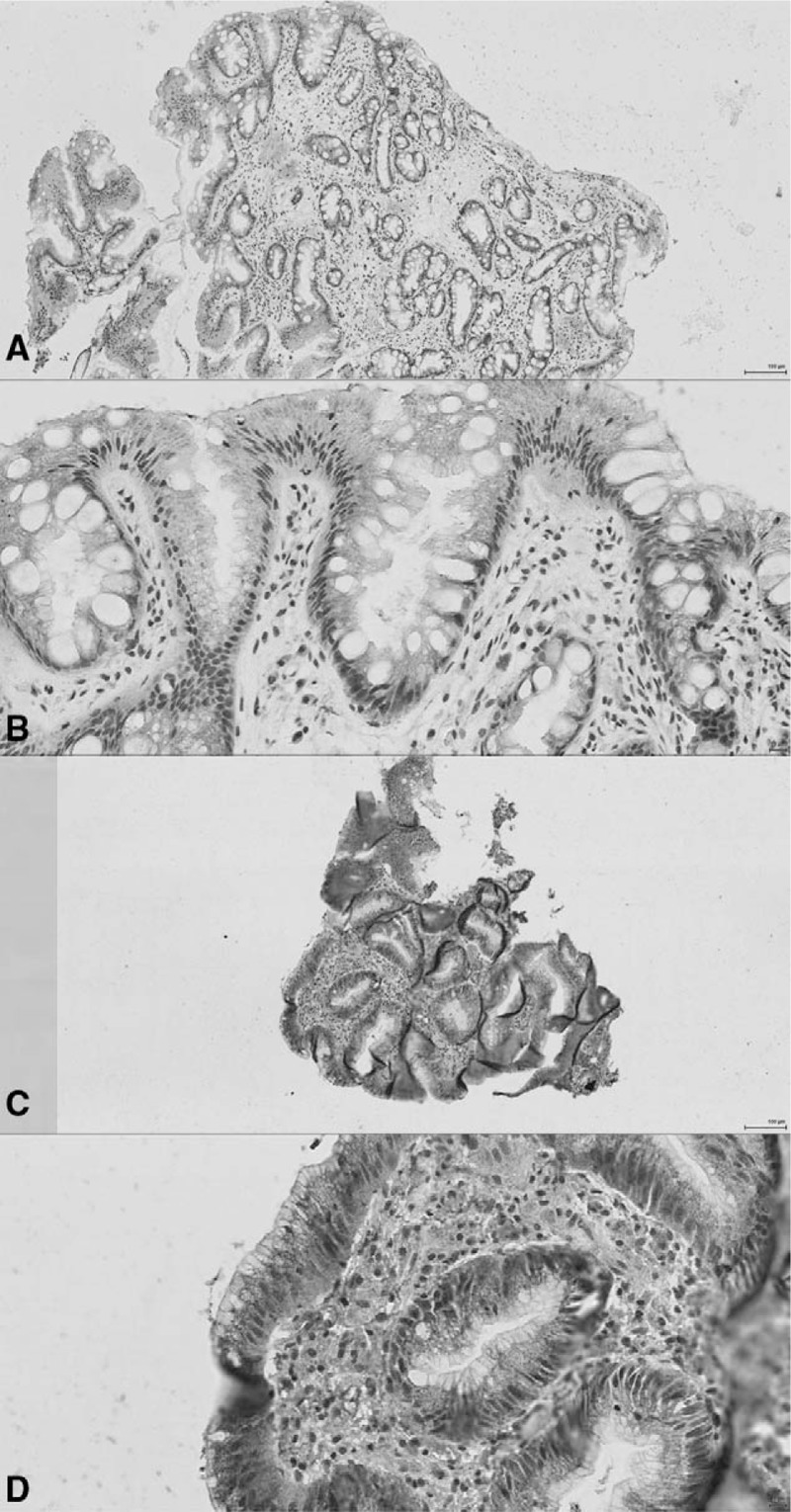
Light micrographs of representative examples of expression of OR3A4 via IHC of nonprogressor (A 10× view, B 40× view) and progressor (C 10× view, D 40×) view.

We then carried out immunohistochemical assessment of expression of OR3A4 (Fig. 4), which, although is labeled as long noncoding RNA, is actually expressed in tissues (see Supplementary results), in a subset of 12 patients. For the stromal compartment, a median expression of 6 (IQR 4–7) was seen in progressors and 2 (IQR 1–3) in nonprogressors (Wilcoxon rank-sum *P* = 0.0308, *z* = −2.160). For the epithelial compartment, a median expression of 8 (IQR 8–10) was seen in progressors and 5.5 (IQR 3–10) in nonprogressors (Wilcoxon rank-sum *P* = 0.4587, *z* = −0.741). Percentage methylation at OR3A4 and stromal expression was strongly negatively correlated (Pearson correlation coefficient −0.85, *P* = 0.014), and a similar, but nonsignificant correlation was observed with epithelial expression and methylation at OR3A4 (Pearson correlation coefficient −0.40, *P* = 0.373).

### Ability of OR3A4 Methylation to Act as a Discriminator in BE

To understand the accuracy of using methylation within cg09890332 of OR3A4 as a biomarker for high-risk BE, we carried out a multivariable reverse stepwise logistic regression analysis of methylation at the 3 tagged CpG dinucleotides within the pyrosequencing assay as the independent variables and progressor versus nonprogressor status as the dependent variables. In this model, CpGs 2 and 3 became nonsignificant (*P* = 0.3325 and *P* = 0.4764) and were removed from the model, leaving the first CpG in cg09890332 as being significant [coef = −0.0563, SE = 0.016, *z* = −3.40, *P* = 0.001, 95% confidence interval (CI) −0.089 to −0.024]. Using receiver-operator curve modeling, the area under curve of this model was 0.82 (95% CI 0.80–0.83) in the cohort where the marker was originally generated (Supplementary Fig. 2).

We then used the *diagt* function of Stata 11.2 to model a set methylation threshold effect on sensitivity and specificity of the test, aiming for maximum negative predictive value and correcting for an incidence rate within the cohort of 0.7%. Modeling at a threshold of below 89% being significant showed that hypomethylation at OR3A4 can predict progression to invasive carcinoma with a sensitivity of 70.8%, specificity of 86%, positive predictive value of 85% and negative predictive value of 72.5%.

We then carried out validation bisulfite pyrosequencing on a cohort of progressors (n = 18) versus nonprogressors (n = 14, Southampton cohort), finding that there were significant differences (*P* = 0.0477, unpaired *t* test) in methylation, with an average methylation of 59.2% (95% CI 56.2%–62.1%) in progressors versus 63.5% (95% CI 60.2%–66.7%) in the nonprogressors. Regression model demonstrated area under curve 0.70, and adjustment for a prevalence of 0.7% using a threshold of 58% demonstrated a sensitivity of 33.3%, specificity of 78.6%, positive predictive value of 10.5%, and negative predictive value of 94%.

## CONCLUSIONS

We have identified that hypomethylation at cg09890332 corresponding to the CG nucleotide at position (CHR) of *OR3A4* can discriminate between patients who progress from nondysplastic BE and those who did not. This association is maintained across independent cohorts, and seems to be related temporally (ie, the association is maintained in the earliest set of samples from a time series of follow-up biopsies in patients with BE), and also by case status. Gastroesophageal reflux is a key risk factor in the development of BE,^19^ and also obesity and cigarette smoking.

The effect of hypomethylation on the *OR3A4* gene seems to be functional, in that immunohistochemistry reveals an increase in *OR3A4* expression in samples with hypomethylation. The finding that the stromal expression, in particular, is increased is of interest, given the known effect of “pathological” stroma in the pathogenesis of esophageal cancer.^37^ Our observed region coincides within 225 bp of a CTCF and RAD21 transcription factor binding site, further suggesting that methylation there has a functional effect to prevent transcription factor binding and alter gene expression. Guo et al^38^ performed a genome-wide screen of long noncoding RNAs in gastric adenocarcinoma, finding that *OR3A4* was significantly (55.9-fold) overexpressed in these patients. They also observed that levels of *OR3A4* were correlated with metastatic potential and prognosis. Furthermore, they utilized *OR3A4* overexpression vectors and performed siRNA knockdown to demonstrate that *OR3A4* seems to regulate cellular proliferation in gastric cancer cell lines. Finally, they utilized their overexpressing cell line models and implanted them into nude mice, finding that *OR3A4* overexpressing gastric cancer cell lines grew significantly faster and more aggressively than with knockdown of *OR3A4*. Downstream analysis of target genes demonstrated that *OR3A4* targets *PDLIM2*, a putative tumor suppressor than regulates cell cycle and adhesion; *PIWIL1*, a transcriptional silencer; and *DLX4* which induces epithelial-mesenchymal transition via *TWIST1*.

We found both at the individual probe level and as part of a DMR that there is hypomethylation in the CpG island associated with *FGFR2*; however, this did not validate at the single probe level when examined with bisulfite pyrosequencing. *FGFR2* has been observed to undergo recurrent alteration in both esophageal adenocarcinoma^39^ and squamous cell carcinoma,^40^ with the latter demonstrating recurrent amplification. The disparity between our microarray results and validation by pyrosequencing may be due to probe inflation caused by small sample size, and is a significant weakness of our study; however, given its biological associations with esophageal adenocarcinoma, further work is needed.

In common with premalignant lesions in cancer, such as colorectal adenomatous polyps,^30^ we observed a trend towards genome-wide hypomethylation as demonstrated by a leftward shift of our genome-wide volcano plot, suggesting a widespread overexpression of genes as part of the development towards malignancy. We also found no difference in chromosomal instability between progressors and nonprogressors, although there was widespread instability within both sets of samples, in common with what has previously been observed^41^ in BE.

Another weakness of our study was the inability to carry out a more comprehensive validation of all observed markers as part of a larger panel of markers. Our study made use of extremely small tissue biopsies from endoscopic surveillance programs, which limited the quantity of usable DNA that could be extracted from these samples and used for downstream validation, and thus validation of the observed DMR and other differentially methylated position regions could not be carried out. We were also limited in the number of samples that could be tested, because of the rarity of biopsy samples before the diagnosis of esophageal cancer, as we took advantage of a local screening program to obtain samples. However, in both the genome-wide and in the validation phase, we believe we have sufficient power to detect methylation changes in this marker. In the genome-wide phase, our sample size of 24 patients would allow us to detect a methylation difference^42^ of 2%, with a statistical power of 90%. Similarly, in the validation phase, our sample size would allow us to detect a minimum methylation change of 10% in the sample set, given the previously observed median methylation and standard deviation in these samples.

We observed a median time to diagnosis of esophageal adenocarcinoma on commencement of the surveillance program of 114 months, which we believe is a reflection of the early identification of these patients and their enrolment into a screening program, and the known slow progression of esophageal adenocarcinoma. A further problem with molecular genetic analysis is heterogeneity, due to the low proportion of cells within a biopsy specimen that contain changes compatible with BE, which leads to less clear methylation changes.

Hypomethylation of *OR3A4*, although seemingly accurate for the detection of progression of Barrett to invasive adenocarcinoma, is likely to be of more utility as a multimodal stratifier in BE, taking account of previous findings at the mutational and copy number level, and also epigenetic change. However, for the purpose of designing a surveillance program with the ability to risk-stratify the nondysplastic BE patient, this marker has significant potential utility.

Development of a streamlined surveillance program could lead to cost savings through the avoidance of unnecessary upper gastrointestinal endoscopy or via a less invasive technology such as the CytoSponge,^43^ in a low-risk cohort identified by a molecular marker panel. More frequent endoscopy in the high-risk cohort could lead to earlier diagnosis of EADC or initiate management of BE to arrest further progression. In conclusion, development of a stratified marker panel in the context of a clinical trial is now needed to improve diagnosis of high-risk BE.

## Supplementary Material

Supplemental Digital Content
