# Supplementary material for: Whole Genome Methylation Analysis of Nondysplastic Barrett Esophagus that Progresses to Invasive Cancer
Source: Ann Surg. 2018 Jan 30;269(3):479–85. doi: 10.1097/SLA.0000000000002658 (PMC6369874; doi:10.1097/SLA.0000000000002658)
Supplement: Supplemental Digital Content [file ansu-269-479-s001.docx]

Supplementary methods for Dilworth et al

**Methylation analysis of microarray data**

The ChAMP package for methylation array analysis was used to analyse methylation data. The pipeline consists of several steps. Firstly, raw data in the form of Illumina IDAT files were imported from the slide scans using a wrapper for the *minifi* package. Filtering of probe level data is carried out by removing all probes with a detection p-value > 0.01, then removing probes with < 3 beads in at least 5% of the samples of the probe, then non-CpG probes are filtered out, then SNP-related probes (PMID 27924034) are filtered, followed by multi-hit probes (PMID 24063430) and finally all X/Y chromosomal probes are removed.

Quality control was performed by examining MDS plots and highlighting samples that did not cluster (add figure in here). SWAN normalisation on probes was then carried out, followed by Singular Value Decomposition analysis (SVD) to identify components of variation. This identified “chip” (as a batch effect of the use of multiple chips) and “Sample Group” suggesting there were significant variations between progressors vs. non-progressors. Batch effects were then corrected for using COMBAT, followed by calling for methylation variable positions.

Methylation variable positions were called for using champ.DMP() function which implements the limma package to calculate the p-value for differential methylation using a linear model, in a case-vs-control fashion. Differentially methylated regions were called for using the champ.DMR function and the ProbeLasso subfunction. Copy number calling was performed by the copy number calling function of ChAMP.

**Scoring of IHC**

IHC was scored blinded by two independent reviewers, one of which was an NHS Consultant Histopathologist. Scoring was divided into nuclear, cytoplasmic and membranous in the epithelial and stromal components separately, on a score of 1-4 where 1=negative, 2=weak positive, 3=positive and 4=strong positive.

**Supplementary results**

In order to explore the expression of OR3A4 at the mRNA level given its’ described function as a long non-coding RNA, we carried out a search of the GTEx Portal (<https://gtexportal.org>) searching for the gene ID ENSG00000262670.1. The results are shown in supplementary figure X, showing that it is highly expressed in testis and at a lower level in other tissues.

At the protein level a search of the Protein Proteomics DB (<https://www.proteomicsdb.org> ) revealed that OR3A4 (P47883) was expressed at the protein level across multiple experiments including the proteome of the MCF-7 cell line, retinal stem cells and placenta suggested that it may undergo transcription, at least partially.

Supplementary Figure 1: Box plot of RPKM values for OR3A4 expression at mRNA level across multiple tissue types taken from RNAseq data from the Gene Expression Omnibus (GTEx)


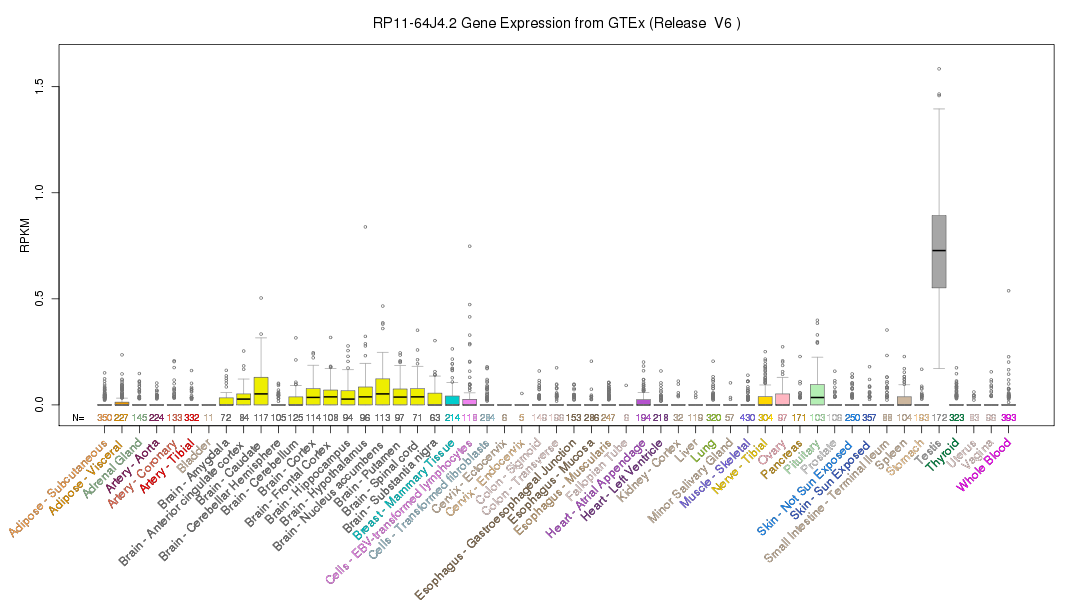


**Supplementary figure 2: ROC curve demonstrating the ability of *OR3A4* methylation to differentiate high risk BO**
